# Deletion of Bmal1 Impairs Pancreatic *β*-Cell Function via Mitochondrial Signaling Pathway

**DOI:** 10.1155/2020/9803024

**Published:** 2020-09-07

**Authors:** Lu Ye, Huaxiang Wu, Weihong Xu

**Affiliations:** Department of Rheumatology, School of Medicine, The Second Affiliated Hospital of Zhejiang University, No. 88, Jiefang Road, Hangzhou 310009, China

## Abstract

Several studies have demonstrated that brain and muscle Arnt-like protein-1 (Bmal1) acts as a core clock gene for maintaining normal cell function, including hepatocytes and cardiomyocytes. Loss of Bmal1 is associated with type 2 diabetes due to pancreatic *β*-cell failure. However, little information is available about its role and mechanism in pancreatic *β*-cell. To address this, we investigated the consequences of Bmal1 inhibition in an insulinoma cell line (INS-1) by using small interfering RNA (siRNA). We observed that knockout of Bmal1 impaired glucose-stimulated insulin secretion in *β*-cell. Meanwhile, the depletion of Bmal1 in *β*-cell caused an adverse change in mitochondrial membrane potential and mitochondrial architecture. Deletion of Bmal1 attenuated mRNA and protein expression of mitofusin 1 (Mfn1) and mitofusin 2 (Mfn2) and enhanced the expression of fission 1 (Fis1). In summary, the deletion of Bmal1 impaired *β*-cell function may be via the mitochondrial signaling pathway in INS-1 cells.

## 1. Introduction

Type 2 diabetes, a disease characterized by hyperglycaemia, has become one of the major chronic diseases that seriously endanger human health. Pancreatic *β*-cell dysfunction is a key factor in the development of type 2 diabetes [1]. However, the pathogenesis is not fully understood. Recent studies have shown that circadian clocks have an important role to play in diabetes [2].

Circadian clocks regulate various aspects of behavioral and metabolic processes in mammals [2]. This circadian system, composed of a master clock that locates in the hypothalamic suprachiasmatic nucleus (SCN) and various peripheral tissue clocks, is generated by a feedback loop formed by core clock genes including brain and muscle Arnt-like protein-1 (Bmal1), circadian locomotor output cycles kaput (Clock), Period (Per), and Cryptochrome (Cry). Bmal1 and Clock activate the transcriptional process of Per and Cry, while Per and Cry inhibit Clock/Bmal1 transactivation activity [3]. Interestingly, previous animal and human studies have linked abnormal Bmal1 expression to metabolic disease. For example, disruption of Bmal1 leads to diabetes, obesity, abnormal gluconeogenesis, and lipogenesis [4–6]. Moreover, certain haplotypes of the Bmal1 gene have been demonstrated to be associated with an increased risk of diabetes and hypertension in human studies [7, 8]. Bmal1 depletion in mice enhances very-low-density lipoprotein (VLDL) assembly and secretion with the downregulation of src homology-2 domain-containing protein tyrosine phosphatase (Shp) and the upregulation of microsomal triglyceride transfer protein (Mtp). Meanwhile, Bmal1 deletion inhibits cholesterol secretion to the bile with the downregulation of Gata-binding protein 4 (Gata4) and ATP-binding cassette family G protein 5 and protein 8 (Abcg5 and Abcg8) [9]. Bmal1 regulates autophagy in cardiomyocytes via the mammalian target of the rapamycin (mTOR) signaling pathway [10]. Bmal1 knockout mice develop obesity [11]. However, the mechanism of Bmal1 in pancreatic *β*-cell is not well elucidated.

Mitochondria are required for normal *β*-cell function and are known to be responsible for cellular metabolism including adenosine triphosphate (ATP) production and cell apoptosis. Mitofusin 1 (Mfn1), mitofusin 2 (Mfn2), and fission 1 (Fis1), core mitochondrial dynamics proteins that localized on the mitochondrial outer membrane, are known as key regulators of mitochondrial networks [12–15]. It has been reported that mitochondrial dysfunction could impair glucose utilization and insulin secretion in *β*-cells [12, 16]. Intriguingly, mitochondrial respiration displays daily oscillations [17]. Moreover, circadian clocks have been reported to regulate mitochondrial acetylation and various essential proteins involved in metabolic pathways [18]. Furthermore, mitochondrial deacetylation is demonstrated as Bmal1 dependent [19]. These data indicate that mitochondrial function is under circadian clock control.

In the present study, we evaluated the function of Bmal1 in INS-1 cells by using small interfering RNA (siRNA). We investigated whether Bmal1 could influence normal *β*-cell function and apoptosis. Our results suggest that the deletion of Bmal1 could impair *β*-cell function via the mitochondrial signaling pathway.

## 2. Materials and Methods

### 2.1. Materials

Rat insulinoma cell line (INS-1) was from the American Type Culture Collection (Manassas, VA, USA). Rabbit anti-Bmal1 antibody was purchased from Abcam (Cambridge, MA, USA). Rabbit anti-Mfn1, anti-Mfn2, anti-Fis1, and anti-GAPDH antibodies were from Proteintech (Chicago, IL, USA). RPMI-1640 medium and cell culture reagents were from Gibco (Grand Island, NY, USA).

### 2.2. Cell Culture

INS-1 cells were grown in a monolayer culture in RPMI-1640 medium containing 10% fetal bovine serum, 2 mM L-glutamine, 1 mM sodium pyruvate, 50 *μ*M *β*-mercaptoethanol, 10 mM 4-(2-hydroxyethyl)-1-piperazineethanesulfonic acid, 100 U/mL penicillin, and 100 *μ*g/mL streptomycin, with a humidified atmosphere of 5% CO_2_ and 95% air at 37°C. Cell passage was performed when the cells reached 70% confluency.

### 2.3. Cell Transfection

The negative control siRNA was obtained from RiboBio (Guangzhou, China). Then, siRNA was introduced into *β*-cell by using Lipofectamine™ 2000 Reagent (Invitrogen, Carlsbad, CA, USA).

### 2.4. Quantitative Real-Time PCR

Total RNA was extracted by using Trizol reagent according to the manufacturer's guidelines. Then, total RNA was reverse transcribed by using HiScript Reverse Transcriptase (RNase H) (Vazyme, Piscataway, NJ, USA). Quantitative PCR (qPCR) was carried out on equal amounts of cDNA in triplicate for each sample using the Power SYBR Green PCR Master Mix (Vazyme, Piscataway, NJ, USA). The GAPDH gene was used as an internal control. The mRNA expression levels were calculated by the 2^-*ΔΔ*Ct^ method and normalized to GAPDH expression. Primer sequences are listed in Table 1.

### 2.5. Western Blot Analysis

The protein expression levels of Mfn1, Mfn2, and Fis1 were determined by western blot. Pancreatic *β*-cell proteins from each group were extracted in iced cell lysis buffer (Beyotime Institute of Biotechnology, Haimen, China). Protein samples were separated by 10% sodium dodecyl sulfate-polyacrylamide gel electrophoresis (SDS-PAGE) and then transferred to polyvinylidene difluoride (PVDF) membrane. Samples were probed with primary antibodies and then incubated with secondary antibodies. The signals were detected with the ECL reagents (Applygen, Haidian, Beijing, China), and relative intensities of protein bands were analyzed by BandScan software.

### 2.6. Measurement of Mitochondrial Membrane Potential

The mitochondrial membrane potential (MMP) in *β*-cells was determined with the JC-1 assay kit (Beyotime Institute of Biotechnology, Haimen, China). The fluorescence intensity of mitochondrial JC-1 monomers and aggregates was determined with flow cytometry (Beckman Coulter, Miami, FL, USA). The JC-1 aggregates which emitted red fluorescence were at high MMP, while the JC-1 monomers which showed green fluorescence were at low MMP.

### 2.7. Transmission Electron Microscopy

Samples were fixed with glutaraldehyde for 2 h and then with osmium tetroxide for 1 h. Subsequently, samples were dehydrated in acetone and embedded in SPI-Pon-812. Sections were sliced into 0.1 mm by a thin-sliced cutting machine, then stained with lead citrate and uranyl acetate, and then observed by a HT7700-SS electron microscope (Hitachi, Tokyo, Japan).

### 2.8. Tunel Assay

To further measure apoptosis, a tunel assay was performed. INS-1 cells were fixed in 4% paraformaldehyde for 25 min, washed in PBS, and then permeabilized with 0.1% Triton X-100 (Beyotime Institute of Biotechnology, Haimen, China) for 10 min on ice. Using a tunel assay for 60 min at 37°C according to the manufacturer's protocol (Roche Applied Science, Basel, Switzerland), the images were captured under a fluorescence microscope (Olympus, Tokyo, Japan), and the tunel-positive cells were counted. Additionally, DAPI (Beyotime Institute of Biotechnology, Haimen, China) was used to stain the cell nuclei.

### 2.9. Glucose-Stimulated Insulin Secretion (GSIS)

INS-1 cells were washed with Krebs-Henseleit bicarbonate (KHB), incubated in KHB for 1 hour, and then exposed to 2.8 or 16.7 mmol/L glucose for 1 hour. The supernatant was collected and detected for insulin concentration using the ELISA method.

### 2.10. Statistical Analysis

All values were presented as mean ± SE. Statistical differences were assessed by Student's *t*-test. A *p* value < 0.05 was considered statistically significant. Statistical analyses were performed with SPSS 16.0 (SPSS Inc., Chicago, IL, USA).

## 3. Results

### 3.1. Bmal1 Deficiency Impaired Glucose-Stimulated Insulin Secretion

**Figure 1:**
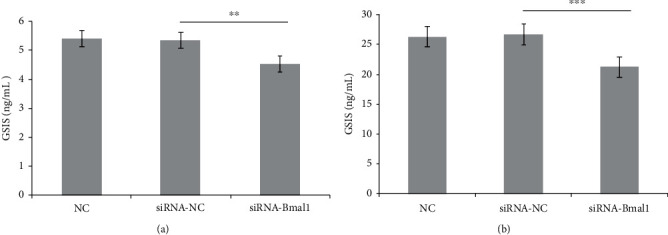
Bmal1 deficiency impaired glucose-stimulated insulin secretion in INS-1 cells. INS-1 cells were incubated with or without Bmal1 inhibition. Insulin concentration was detected by the ELISA method. (a) Presented the mean insulin secretion in response to 2.8 mmol/L glucose. (b) Presented the mean insulin secretion in response to 16.7 mmol/L glucose. NC: normal control group; siRNA-NC: INS-1 cells treated with negative control siRNA; siRNA-Bmal1: INS-1 cells treated with Bmal1 siRNA. A *p* value < 0.05 is considered as statistically significant. ^∗^*p* < 0.05, ^∗∗^*p* < 0.01, and ^∗∗∗^*p* < 0.001.

To investigate the effect of Bmal1 on *β*-cell function, we performed a loss of function experiment. As shown in Figure 1, after the deletion of Bmal1, the mean insulin secretion in response to 2.8 mmol/L glucose was significantly decreased (*p* < 0.01). Moreover, insulin secretion in response to 16.7 mmol/L glucose was also significantly reduced. Therefore, Bmal1 is required for normal *β*-cell function (*p* < 0.001).

### 3.2. Bmal1 Deficiency Increased Cell Apoptosis

**Figure 2:**
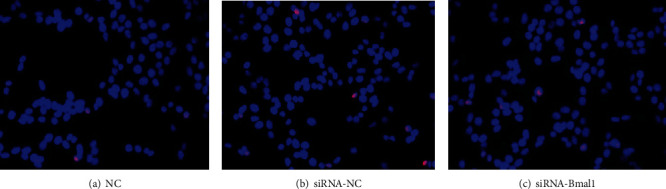
Bmal1 deficiency increased cell apoptosis of INS-1 cells. INS-1 cells were incubated with or without Bmal1 inhibition. Apoptotic cells were detected by the tunel assay. Images were obtained at magnifications of 400x. The nucleus of normal cells was blue and of apoptotic cells was pink. NC: normal control group; siRNA-NC: INS-1 cells treated with negative control siRNA; siRNA-Bmal1: INS-1 cells treated with Bmal1 siRNA.

To investigate the effect of Bmal1 on cell apoptosis, INS-1 cells were cultured in a normal glucose medium, with or without Bmal1 inhibition. Apoptotic cells were detected by the tunel assay. As shown in Figure 2, although not statistically significant, the total percentage of apoptotic cells had a trend to increase in cells lacking Bmal1 (*p* > 0.05).

### 3.3. Bmal1 Deficiency Attenuated Mitochondrial Membrane Potential

**Figure 3:**
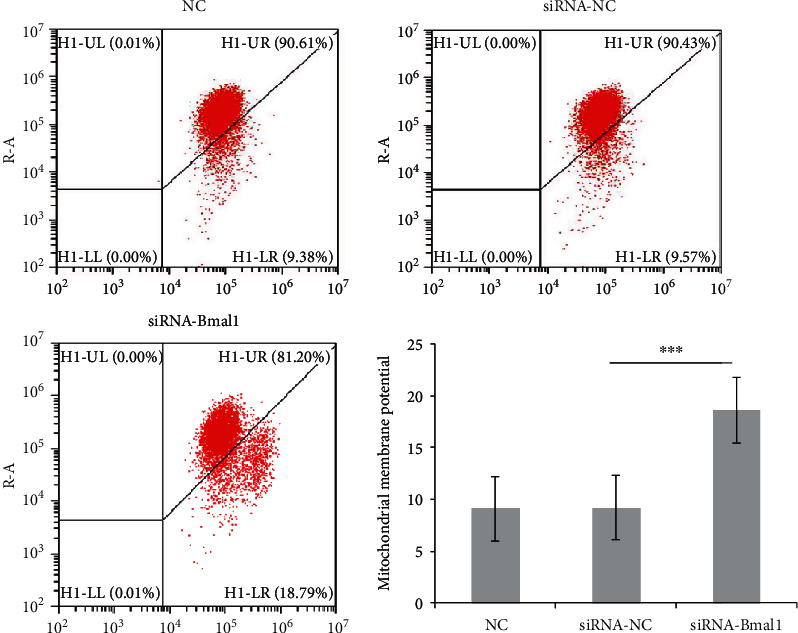
Bmal1 deficiency decreased mitochondrial membrane potential. The upper right quadrant (UR) indicates that JC-1 is present in the aggregative form, which exhibits red fluorescence with high mitochondrial membrane potential. The lower right quadrant (LR) indicates that JC-1 maintains in the monomeric form, which emits green fluorescence with low mitochondrial membrane potential. INS-1 cells were treated with or without Bmal1 inhibition after loading with JC-1 dye. The representative images were shown above. NC: normal control group; siRNA-NC: INS-1 cells treated with negative control siRNA; siRNA-Bmal1: INS-1 cells treated with Bmal1 siRNA. A *p* value < 0.05 is considered as statistically significant. ^∗^*p* < 0.05, ^∗∗^*p* < 0.01, and ^∗∗∗^*p* < 0.001.

MMP is an essential indicator of cellular metabolic activity. In addition, ROS, a core molecule related to oxidative stress, is generated by the potential difference across the inner mitochondrial membrane [20]. To investigate the effect of Bmal1 on MMP, we examined the alteration of MMP in *β*-cells with or without Bmal1 inhibition by the JC-1 assay. As shown in Figure 3, in comparison with the normal control group, the intensity of green fluorescence emitted by the JC-1 monomer increased in cells treated with Bmal1 siRNA (*p* < 0.001), indicating that MMP was decreased. This result indicated reduced cellular activity in Bmal1-deficient *β*-cells.

### 3.4. Bmal1 Deficiency Altered Mitochondrial Architecture

**Figure 4:**
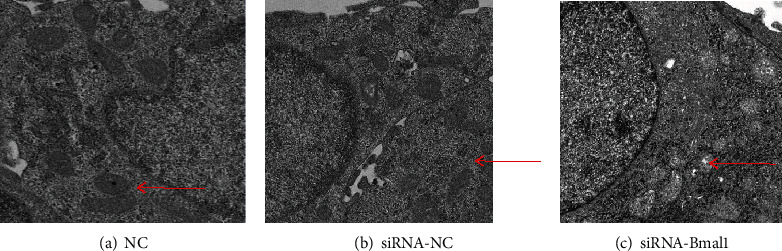
Bmal1 deficiency altered mitochondrial architecture in *β*-cells. Images were obtained at magnifications of 5000x. (a, b) Most mitochondria were round or oval with intact membrane and a few tubular and vesicular cristae. (b) Most mitochondria were swollen with irregular shape and fracture and disappearance of mitochondrial cristae. NC: normal control group; siRNA-NC: INS-1 cells treated with negative control siRNA; siRNA-Bmal1: INS-1 cells treated with Bmal1 siRNA.

To investigate the effect of Bmal1 on mitochondrial architecture, we performed transmission electron microscopic analysis. Indeed, pathomorphological changes in the mitochondria of Bmal1 knockdown cells were observed by transmission electron microscopy. As shown in Figure 4, in the control group, most mitochondria were round or oval with intact membrane and a few tubular and vesicular cristae, whereas, in Bmal1-deficient *β*-cells, mitochondria were swollen with irregular shape and fracture and disappearance of mitochondrial cristae.

### 3.5. Bmal1 Deficiency Altered Mitochondrial Fusion and Fission Genes

**Figure 5:**
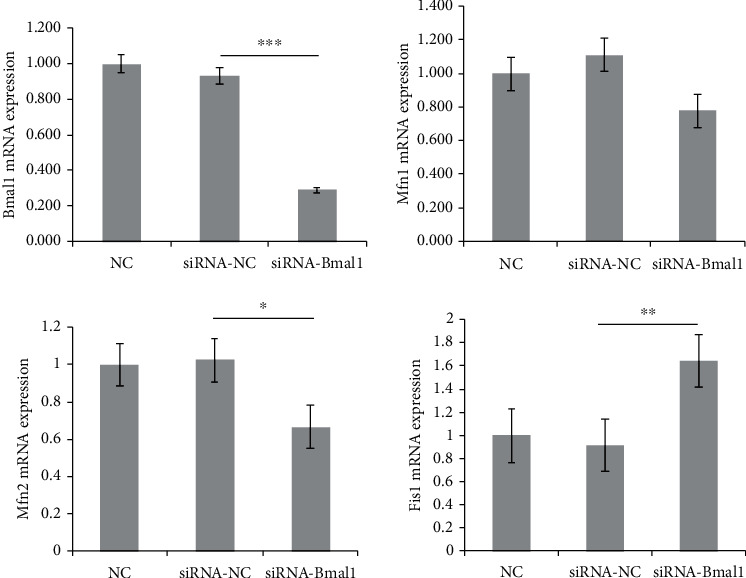
Bmal1 deficiency in *β*-cells altered core genes related to mitochondrial dynamics. Bmal1, mitofusin 1, mitofusin 2, and fission 1 mRNA were analyzed by qPCR. Expression levels were normalized to GAPDH mRNA, and each bar represents the mean ± SE of 3 individual experiments. NC: normal control group; siRNA-NC: INS-1 cells treated with negative control siRNA; siRNA-Bmal1: INS-1 cells treated with Bmal1 siRNA. A *p* value < 0.05 is considered as statistically significant. ^∗^*p* < 0.05, ^∗∗^*p* < 0.01, and ^∗∗∗^*p* < 0.001.

**Figure 6:**
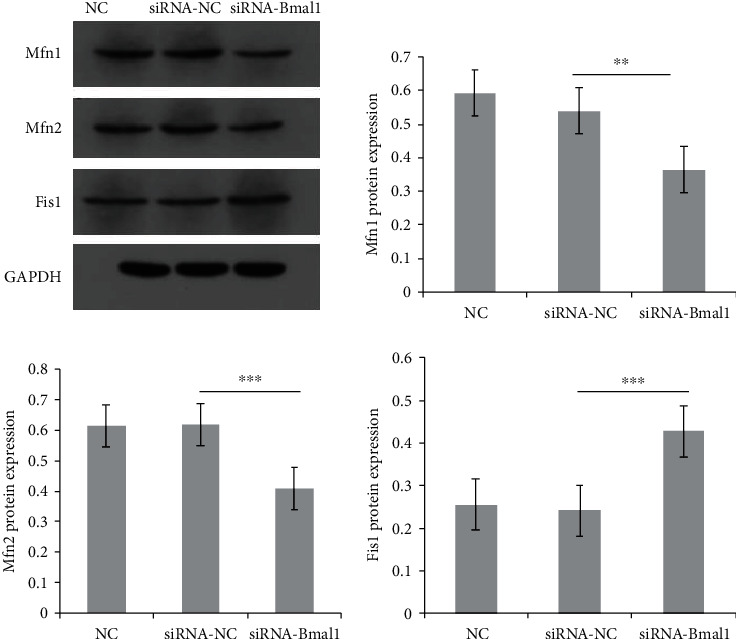
Bmal1 deficiency in *β*-cells altered essential proteins related to mitochondrial dynamics. Mitofusin 1, mitofusin 2, and fission 1 protein expressions were analyzed by western blot. Expression levels were normalized to GAPDH protein expression, and each bar represents the mean ± SE of 3 individual experiments. NC: normal control group; siRNA-NC: INS-1 cells treated with negative control siRNA; siRNA-Bmal1: INS-1 cells treated with Bmal1 siRNA. A *p* value < 0.05 is considered as statistically significant. ^∗^*p* < 0.05, ^∗∗^*p* < 0.01, and ^∗∗∗^*p* < 0.001.

To investigate the effects of Bmal1 on mitochondrial dynamics in *β*-cell, we knocked down Bmal1 expression in INS-1 cells by using siRNA. After transfection with Bmal1 siRNA, Bmal1 mRNA expression was significantly reduced by 71.1% compared with that in the control group (*p* < 0.001) as shown in Figure 5. We then examined core molecules associated with mitochondrial dynamics and quality control including Mfn1, Mfn2, and Fis1 by quantitative real-time PCR. Interestingly, as shown in Figure 5, gene expression changes were observed in Bmal1-deficient *β*-cells. Mfn1 mRNA expression showed an obvious decrease trend (*p* = 0.07). Mfn2 mRNA expression was significantly reduced (*p* < 0.05), while Fis1 was remarkably increased (*p* < 0.01). Similar results were observed in western blot analysis. As shown in Figure 6, protein expression of Mfn1 and Mfn2 was obviously decreased, while Fis1 was significantly elevated (*p* < 0.01, *p* < 0.001, and *p* < 0.001, respectively).

## 4. Discussion

The important role of the circadian clock in diabetes and mitochondrial network has been recognized [2, 21]. Mitochondrial proteome, acetylome, lipidome, morphology, and nutrient utilization and respiration exhibit circadian rhythms [21]. However, little is known about the required role of the clock gene Bmal1 in the pancreatic *β*-cell and mitochondrial signaling pathway.

To investigate the role of Bmal1 in *β*-cell function, we performed Bmal1-knockdown experiments. We observed that suppression of Bmal1 impaired glucose-stimulated insulin secretion in *β*-cell. This result is consistent with another previous study [22]. In addition, Bmal1 is a regulator of compensatory *β*-cell expansion and survival in mice [11]. Similar results have been observed in other tissues including myocardial stromal fibroblasts, macrophages, and glioblastoma stem cells [23–25]. These data indicate the important role of Bmal1 in maintaining normal cell function including *β*-cell.

Furthermore, MMP is a key indicator to determine cellular metabolic activity [26]. The present study revealed a decreased MMP in cells lacking Bmal1, indicating decreased *β*-cell function. Additionally, our previous work (data not shown) and another study showed an obvious increase of ROS accumulation and a decrease of ATP production in *β*-cell when inhibiting Bmal1 expression [22]. This is particularly important that ROS clearance and the antioxidant capacity of *β*-cell are much lower than other tissues [27]. Thus, *β*-cell is more sensitive to oxidative stress. Indeed, an increased ROS level could cause deficient GSIS [28]. Moreover, mitochondrial ATP production is critical for glucose-stimulated insulin secretion in *β*-cells [12, 13, 21]. These data indicate that Bmal1 deficiency is closely associated with *β*-cell dysfunction.

In addition, in the present study, ultrastructural changes including swollen mitochondria with irregular shape and cristae damage were observed in Bmal1 knockdown *β*-cell. Similar results have been observed in Bmal1-deficient hepatocytes [29] and cardiomyocytes [30]. Impaired mitochondrial morphology, reduced ATP synthesis, and accumulation of oxidative stress products can further cause cell damage and apoptosis [31]. Indeed, our results showed that *β*-cell apoptosis had a trend to increase in Bmal1-knockdown *β*-cells in comparison with the control group.

However, the underlying mechanism remains unclear, and the role of Bmal1 in mitochondrial dynamics genes in *β*-cell is not studied. In the present study, we identified reduced Mfn1 and Mfn2 expression and increased Fis1 expression as a consequence of Bmal1 deficiency. Mitochondrial dysfunction is known as a core contributor to pancreatic *β*-cell failure in the pathogenesis of diabetes [32]. Indeed, Mfn1 and Mfn2 are required for mitochondrial remodeling and normal mitochondrial metabolism [14, 33]. Cardiomyocytes lacking Mfn1 or Mfn2 result in mitochondrial fragmentation and display a remarkable defect in mitochondrial fusion and mitochondrial structure [34]. Fis1 has been shown to mediate mitochondrial quality control [35] and transmit an apoptosis signal from the mitochondria to the endoplasmic reticulum [36]. Inhibition of hFis1 function prevents mitochondrial fission [37], while overexpression of hFis1 induced mitochondrial fragmentation and impaired glucose-induced ATP, hyperpolarization, and GSIS [15, 16]. Furthermore, pancreatic *β*-cell Mfn2 knockout had a defect in insulin secretion, mitochondrial fragmentation, and bioenergetic dysfunction [16]. Taken together, these data indicated the essential role of Bmal1 in preserving *β*-cell via mitochondrial dynamics genes.

In summary, we showed that defects in the Bmal1 function alter mitochondrial membrane potential and mitochondrial architecture in *β*-cell. Moreover, we revealed that Bmal1 regulated *β*-cell function *via* mitochondrial dynamics-related genes. These data provide new insights into understanding the role of the clock gene in preserving *β*-cell function.

## Figures and Tables

**Table 1:** Primers used for quantitative real-time PCR.

Gene	Primer	Sequence	Size
*GAPDH*	Forward	5′-ACAGCAACAGGGTGGTGGAC-3′	253 bp
	Reverse	5′-TTTGAGGGTGCAGCGAACTT-3′	
*Mfn1*	Forward	5′-TAGCTTCAACTCCTACTGCTCC-3′	178 bp
	Reverse	5′-GTGACAGAGATGAGTTTCCAGC-3′	
*Mfn2*	Forward	5′-GCAGCGGGTTTATTGTCTT-3′	250 bp
	Reverse	5′-GCGGTGCAGTTCATTCTTAT-3′	
*Fis1*	Forward	5′-AAAGAGGAGCAGCGGGATTAT-3′	178 bp
	Reverse	5′-TGCCTACCAGTCCATCTTTCT-3′	
*Bmal1*	Forward	5′-TGAACCAGACAATGAGGGCT-3′	183 bp
	Reverse	5′-TATGCCAAAATAGCCGTCGC-3′	

## Data Availability

The data used to support the findings of this study are available from the corresponding author upon request.
